# Exposure to *Helicobacter pylori* infection in early childhood and the risk of allergic disease and atopic sensitization: a longitudinal birth cohort study

**DOI:** 10.1111/cea.12289

**Published:** 2014-03-26

**Authors:** A Amberbir, G Medhin, W E Abegaz, C Hanlon, K Robinson, A Fogarty, J Britton, A Venn, G Davey

**Affiliations:** 1 Department of Infectious Disease Epidemiology, London School of Hygiene and Tropical MedicineLondon, UK; 2 Division of Epidemiology and Public Health, University of NottinghamNottingham, UK; 3 Aklilu Lemma Institute of Pathobiology, Addis Ababa UniversityAddis Ababa, Ethiopia; 4 Department of Psychiatry, Addis Ababa UniversityAddis Ababa, Ethiopia; 5 Centre for Biomolecular Sciences, University of NottinghamNottingham, UK; 6 Brighton & Sussex Medical School, University of BrightonBrighton, UK

**Keywords:** birth cohort, eczema, Ethiopia, *Helicobacter pylori*, sensitization

## Abstract

**Background:**

An inverse relation between *Helicobacter pylori* infection and allergic disease has been reported by a range of independent epidemiological studies, but evidence from longitudinal studies is scarce.

**Objective:**

We have investigated the effects of *H. pylori* infection on the incidence and prevalence of allergic diseases and sensitization in a low-income birth cohort.

**Methods:**

In 2005/2006, a population-based birth cohort was established in Butajira, Ethiopia, and the 1006 singleton babies born were followed up at ages 1, 3, and 5. Symptoms of allergic disease were collected using the ISAAC questionnaire, allergen skin tests performed, and stool samples analysed for *H. pylori* antigen and geohelminths. Multiple logistic regression was used to determine the independent effects of *H. pylori* measured at age 3 on the incidence of each outcome between ages 3 and 5 years (in those without the outcome at age 3), controlling for potential confounders, and to additionally assess cross-sectional associations.

**Results:**

A total of 863 children were followed up to age 5. *H. pylori* infection was found in 25% of the children at both ages 3 and 5, in 21% at age 5 but not 3, and in 17% at age 3 but not at age 5. *H. pylori* infection at age 3 was significantly associated with a decreased risk of incident eczema between ages 3 and 5 (adjusted OR, 95% CI, 0.31; 0.10–0.94, *P* = 0.02). Cross-sectionally at age 5, *H. pylori* infection was inversely associated with skin sensitization (adjusted OR, 95% CI, 0.26; 0.07–0.92, *P* = 0.02).

**Conclusion and clinical relevance:**

These findings provide further evidence to suggest that early-life exposure to *H. pylori* may play a protective role in the development of allergy.

## Introduction

There is wide geographical variation in the prevalence of asthma and allergic conditions world-wide, with substantial differences seen between low- and high-income countries, and between urban and rural communities [1–3]. Our group's previous work in Ethiopia has found a threefold higher prevalence of asthma symptoms in urban compared with rural areas [2]. This observation raises the hypothesis that environmental factors, possibly interacting with genetic determinants, or other factors related to urban–rural socio-economic disparity, are likely to be important [3,4].

As part of the hygiene hypothesis, much recent interest has focused on the protective role of *Helicobacter pylori* infection in the aetiology of asthma and allergic disease. This has gained support from a range of epidemiological [5–9], epigenetic [10], and animal model studies [11]. Reduced risks of atopy and asthma have been seen in many human studies [5–10,12,13], both in children [5,6,12,14] and adults [7,13]. *H. pylori* is a bacterium which colonizes the gastric mucosa of approximately half the world's population and is the main cause of peptic ulcer disease and gastric adenocarcinoma [15]. The infection is usually established during early childhood, persists life long, and remains asymptomatic in over 85% of cases. It has been suggested that humans have co-evolved with *H. pylori* and are physiologically adapted to be colonized by these bacteria [16]. The prevalence of *H. pylori* infection in developed countries has been declining sharply for several years, however [17]. The proportion of young children becoming infected is now extremely low, most probably due to antibiotic use [18]. Puzzlingly, there has also been a selective decline in the more virulent CagA^+^ strains of *H. pylori* [19]. In opposition to this, between 1973 and 1994, Kosunen et al. [20] found that markers of allergy increased by more than threefold, with the increase mainly occurring in *H. pylori*-negative subjects [20]. These findings are therefore consistent with a secular trend: as *H. pylori* infection has declined [19,20], asthma prevalence has risen [3].

Whilst the role of *H. pylori* in asthma and allergic disease aetiology is intriguing, most of the studies to date are of cross-sectional or case–control design and based in adult high-income country populations, with very few conducted in children. This is important as *H. pylori* is usually acquired in childhood, and its stimulation of the immune system as it develops could be protective [21]. Alternative explanations for the inverse trends, including reverse causation and bias due to antibiotic eradication therapy affecting *H. pylori* acquisition, are difficult to exclude. Therefore, we have used data from our Ethiopian birth cohort followed to age 5, to longitudinally determine the effects of *H. pylori* infections on the incidence of allergic diseases and sensitization, and additionally explore cross-sectional associations at age 5. Cross-sectional associations between *H. pylori* and geohelminth infections and allergic outcomes at age 3 have been reported previously [14]; prevalence of geohelminth infection in this cohort was too low to explore this exposure further.

## Methods

### The study setting and the birth cohort

**Fig. 1:**
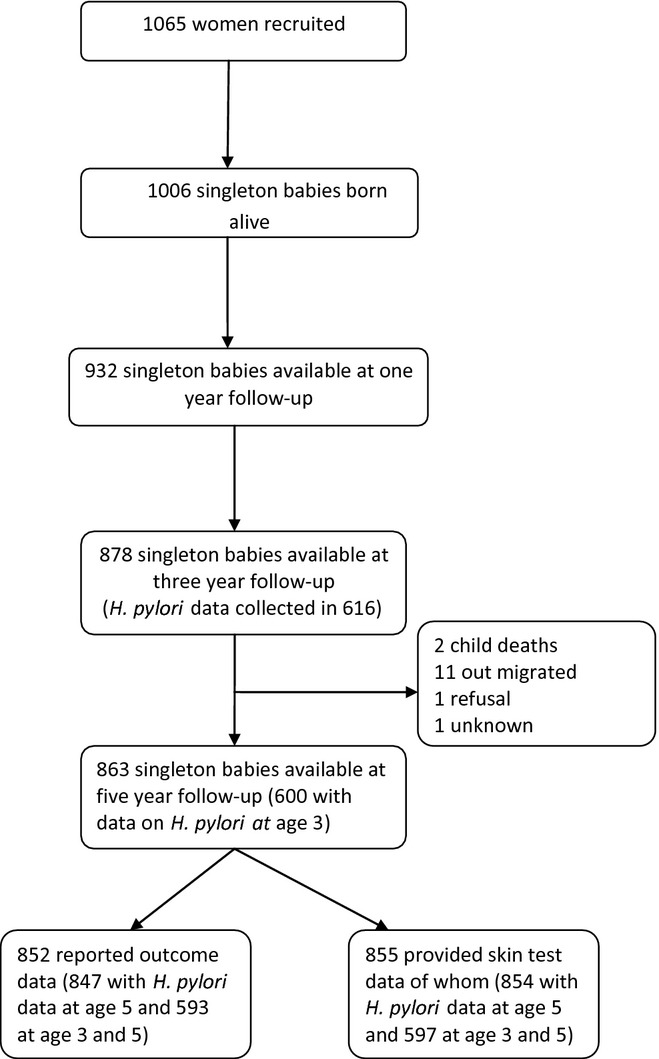
Cohort of children from birth until age 5.

In 2005, a birth cohort was established in Butajira, Ethiopia, details of which have been described previously [22]. In brief, all pregnant women living in the study area were recruited in their third trimester (*N* = 1065) and subsequently gave birth to 1006 singleton live babies who were followed at regular intervals to age 5 (Fig. 1).

### Data collection and measurements

At ages 1-, 3-, and 5-year follow-ups, female data collectors who knew the study setting visited the mothers at home, administered an interview led questionnaire, usually within 2 weeks of the child's birthday. The questionnaire included questions on wheeze, eczema, rhinitis, and asthma based on the International Study of Asthma and Allergies in Children (ISAAC) core allergy and environmental questionnaire as in our previous studies at age 1- [23,24] and 3-year follow-up [14,25]. For wheeze: ‘Has your child ever had (or in the past 12 months) had wheezing or whistling in their chest?' Eczema: ‘Has your child ever had (or in the past 12 months) had an itchy skin rash which has affected the skin creases (e.g. front of the elbow, behind the knees, the front of the ankles, around the neck, or around the eyes?') Rhinitis: ‘Has your child ever had (or in the past 12 months) had problems with sneezing or running nose (when not affected by cold or flu), or problems with itchy watery eyes?’ Information was also collected on a range of potential confounders, including familial factors (maternal and paternal history of asthma and allergy); childhood factors (immunization, breastfeeding status, birth order, number of siblings, child's use of antibiotic); household characteristics (roof type, household size and child's sleeping place); and environmental factors (indoor pollution including indoor cooking, indoor kerosene use, and insecticide use).

At ages 3 and 5, skin sensitization to *D. pteronyssinus* and cockroach allergen (*Blattella germenica*; Biodiagnostics, Upton-upon-Severn, UK), previously found to be common in an Ethiopian population [26], was measured on each child using skin-prick lancets. We demonstrated a reasonably good inter-rater agreement amongst the fieldworkers who did the test (κ = 0.67, *P* < 0.01 for cockroach allergen, and, κ = 0.63, *P* < 0.01 for *D. pteronyssinus* allergen).

Also, a rapid test (Medimar immunocard) was used to determine *H. pylori* antigen in stool samples (Biohit, Unit 1 Barton Hill Way, UK) collected at age 3 from a randomly chosen subsample of children (*N* = 616) and at age 5 from all available children (*N* = 863). Analysis of geohelminth infections was also carried out on the stool samples.

### Data management and statistical analysis

Data were double-entered into EpiData, version 3, cleaned, coded, and merged using Stata 11 (Statacorp, College Station, TX, USA). For analysis of incident wheeze between ages 3 and 5, those children without reported wheeze at ages 1 and 3 (negative response to wheeze in the past 12 months, at years 1 and 3, and wheeze ever at year 3) were selected for analysis and incident wheeze defined as a positive response at the year 5 follow-up. Eczema and rhinitis were analysed in a similar manner. Asthma was very rarely reported in this birth cohort (1%), and therefore not analysed further. For sensitization, those children who were not sensitized at age 3 (sensitization defined as an average of two perpendicular weal diameters, one of which was the maximum measurable diameter, of at least 3 mm greater than the saline control response) were selected for analysis, and new-onset sensitization defined as a positive result at age 5. As the prevalence of cockroach allergen at age 5 was low, a separate analysis of each allergen was not possible; instead, a combined variable ‘any sensitization’ was created to refer to sensitization to either *D. pteronyssinus* or cockroach allergen. For all longitudinal analyses, the exposure variable used was *H. pylori* infection at age 3.

For cross-sectional analysis, the outcomes were wheeze, eczema, and rhinitis in the past 12 months as reported at age 5 and sensitization to either *D. pteronyssinus* or cockroach allergen. The effects of *H. pylori* infection, for all available children at year 5, were analysed by first creating a new exposure variable with categories representing different combinations of infection status at ages 3 and 5: ‘never infected’ (never infected at both time-points), ‘infected at year 3 but not at year 5’, ‘infected at year 5 but not at year 3’, or ‘persistently infected’ (infected at both time-points). For the sensitization outcome at year five, however, numbers of children in some exposure categories were low and it was therefore necessary to merge the exposed categories to create a single category ‘exposed at any age up to year 5’. Moreover, an additional separate analysis of the effects of *H. pylori* infection using only data collected at age five was conducted (infected vs. not infected at age 5), as these analyses are on larger sample sizes than the longitudinal analyses, and benefit from greater statistical power.

Univariate analyses with crude odds ratios (OR) and 95% confidence intervals (CI) for each outcome in relation to *H. pylori* infection were conducted. Multivariate logistic regression was then used to determine the independent effects of *H. pylori* on each incident and prevalent outcome, controlling for the *a priori* confounders, and adjusted ORs, and 95% CIs obtained. The *a priori* confounders were place of residence (urban/rural), child's gender, and maternal education (as a marker of socioeconomic status). The impact of further controlling for any other potential confounders including breastfeeding, antibiotic use, and geohelminth infection was also explored (Tables S1 and S2). These covariates were retained in the model if they have altered the odds ratios for the main exposure of interest by more than 10%. The significance of the association between exposure and outcome in the model was assessed using a likelihood ratio test for the association (LRT).

### Ethics

Ethical approval was granted by the Institutional Review Board (IRB) of Addis Ababa University, the National Ethical Review Committee of the Ethiopian Science and Technology Ministry, and the University of Nottingham, United Kingdom. Written informed consent was obtained from all participants at each study visit.

## Results

### Follow-up of cohort at age 5

Year 1 and 3 follow-ups of the birth cohort have been previously described [14,23]. In brief, 863 singleton children were successfully followed up at year 5 (86% of the original cohort at birth, and 97% of those available at year 3 follow-up), of whom 847 had symptom and *H. pylori* data at this time-point, and 854 had sensitization and *H. pylori* data (Fig. 1). Amongst the wheeze-free cohort at age 3, the incidence of new-onset wheeze (wheeze reported at age 5) was 5.9% (40/676); similarly, the incidence of eczema was 5.8% (39/700), rhinitis 3.9% (31/798), and sensitization (to either *D*. *pteronyssinus* or cockroach allergen) was 2.0% (15/766; Table 1). Sensitization early in life increased the risk of later onset eczema and rhinitis (Table 2). Effects of potential confounders on the outcomes and associations with *H. pylori* at age 5 are found in the online supplement (Tables S1–S3).

**Table 1:** Incidence of the outcomes by area of residence

Incident outcome†	Overall *n* (%)	Urban Yes *n* (%)	Rural Yes *n* (%)	Crude OR (95% CI)	*P*-value
Wheeze (*N* = 676)	40 (5.9)	2 (2.6)	38 (6.4)	0.39 (0.09, 1.64)	0.18
Eczema (*N* = 700)	39 (5.8)	2 (2.4)	37 (6.0)	0.38 (0.09, 1.62)	0.17
Rhinitis (*N* = 798)	31 (3.9)	2 (2.0)	29 (4.2)	0.48 (0.11, 2.03)	0.30
Any sensitization* (*N* = 766)	15 (2.0)	1 (1.1)	14 (2.1)	0.54 (0.07, 4.15)	0.55

* Sensitization to either *D. pteronyssinus* or cockroach allergen.

† Incidence computed in each cohort of children without the outcome at age 3, and incident outcome defined as having the outcome at age 5.

**Table 2:** OR for incident wheeze, eczema, and rhinitis in relation to skin sensitization at the age of three

	Any sensitization at year 3*				
	
New-onset symptoms at age 5	Over all *N* (%)	Yes *n* (%)	No *n* (%)	Crude OR (95% CI)	*P*-value
Wheeze (*N* = 663)	40 (6.0)	5 (8.2)	35 (5.8)	1.45 (0.54, 3.84)	0.46
Eczema (*N* = 688)	39 (5.6)	7 (11.5)	32 (4.9)	2.49 (1.04, 5.95)	0.03
Rhinitis (*N* = 785)	31 (4.0)	6 (8.5)	25 (3.5)	2.54 (1.00, 6.45)	0.04

* Sensitization to either *D*. *pteronyssinus* or cockroach allergen.

### *Helicobacter pylori* infection at ages 3 and 5

*Helicobacter pylori* infection was common with 41% of children infected at age three (described previously [14]) and 44% (377/857) at age five. A pattern of unstable *H. pylori* infection was found such that 17% (102/600) of children were infected at age three but not five, 21% (121/600) at age five but not 3 years, and 25% (147/600) at both ages. Moreover, the prevalence of *H. pylori* infection at age 5 did not significantly differ by urban or rural area of residence such that 50% (52/104) of urban children and 43% (325/753) of rural children have current infection.

### Effects of *H. pylori* infection on incidence of allergic outcomes between ages 3 and 5

In multivariate analysis adjusted for *a priori* confounders, *H. pylori* infection at age 3 was positively, although not significantly related to incident wheeze (adjusted OR, 95% CI, 1.56; 0.69, 3.51, *P* = 0.28; Table 3). Infection, however, was significantly inversely associated with incident eczema, after control for *a priori* confounders and further control for breastfeeding history in the first year of life (adjusted OR, 95% CI, 0.31; 0.10, 0.94, *P* = 0.02; Table 4). Adjustment for other potential confounders made little change to the odds ratio. No significant effects of *H. pylori* were seen on incident rhinitis or sensitization (Table 5 and 6).

**Table 3:** Longitudinal and cross-sectional analyses of wheeze in relation to exposure to *Helicobacter pylori* infection

Longitudinal analysis of incident wheeze between ages 3 and 5 (*N* = 474)*	Overall *N* (%)	Outcome Yes *n* (%)	Outcome No *n* (%)	Crude OR (95% CI)	Adjusted OR† (95% CI)	*P*-value
*H. pylori* exposure at year 3						
No	273 (57.6)	12 (4.4)	261 (95.6)	1	1	0.28
Yes	201 (42.4)	13 (6.5)	188 (93.5)	1.50 (0.67, 3.37)	1.56 (0.69, 3.51)	
Cross-sectional analysis of wheeze at age 5						
*H. pylori* exposure at age 5 (*N* = 847)						
No	477 (56.3)	18 (3.8)	459 (96.2)	1	1	0.35
Yes	370 (43.7)	19 (5.1)	351 (94.9)	1.38 (0.71, 2.67)	1.37 (0.71, 2.65)	
*H. pylori* exposure at ages 3 and 5 (*N* = 593)†						
Never infected	222 (37.4)	10 (4.5)	212 (95.5)	1	1	0.52
Infected at age 3 but not at age 5	101 (17.0)	3 (3.0)	98 (97.0)	0.65 (0.17, 2.42)	0.62 (0.16, 2.30)	
Infected at age 5 but not at age 3	126 (21.3)	5 (4.0)	121 (96.0)	0.88 (0.29, 2.63)	0.89 (0.30, 2.68)	
Persistent exposure	144 (24.3)	10 (6.9)	134 (93.1)	1.58 (0.64, 3.91)	1.52 (0.61, 3.77)	

* Reduced numbers as longitudinal analysis performed only on those reporting never wheeze at age 3.

† ORs adjusted for child's gender, area of residence, and maternal education.

‡ Reduced numbers as *H. pylori* measured on subsample only at age 3.

**Table 4:** Longitudinal and cross-sectional analyses of eczema in relation to exposure to *Helicobacter pylori* infection

Longitudinal analysis of incident eczema between ages 3 and 5 (*N* = 498)*	Overall *N* (%)	Outcome Yes *n* (%)	Outcome No *n* (%)	Crude OR (95% CI)	Adjusted OR† (95% CI)	*P*-value
*H. pylori* exposure at year 3						
No	293 (58.8)	18 (6.1)	275 (93.9)	1	1	0.02
Yes	205 (41.2)	5 (2.4)	200 (97.6)	0.38 (0.14, 1.05)	0.31 (0.10, 0.94)		
Cross-sectional analysis of eczema at age 5						
*H. pylori* exposure at age 5 (*N* = 847)						
No	477 (56.3)	24 (5.0)	453 (95.0)	1	1	0.19
Yes	370 (43.7)	12 (3.2)	358 (96.8)	0.63 (0.31, 1.28)	0.63 (0.31, 1.28)	
*H. pylori* exposure at ages 3 and 5 (*N* = 593)‡						
Never infected	222 (37.4)	15 (6.8)	207 (93.2)	1	1	0.16
Infected at age 3 but not at age 5	101 (17.0)	2 (2.0)	99 (98.0)	0.28 (0.06, 1.26)	0.29 (0.06, 1.28)	
Infected at age 5 but not at age 3	126 (21.3)	5 (4.0)	121 (96.0)	0.57 (0.20, 1.61)	0.54 (0.19, 1.54)	
Persistent exposure	144 (24.3)	4 (2.8)	140 (97.2)	0.39 (0.13, 1.22)	0.40 (0.13, 1.24)	

* Reduced numbers as longitudinal analysis performed only on those reporting never eczema at age 3.

† ORs adjusted for child's gender, area of residence, and maternal education and further adjusted controlled for history of breastfeeding in the first year of life.

‡ Reduced numbers as *H. pylori* measured on subsample only at age 3.

**Table 5:** Longitudinal and cross-sectional analyses of rhinitis in relation to exposure to *Helicobacter pylori* infection

Longitudinal analysis of incident rhinitis between ages 3 and 5 (*N* = 559)*	Overall *N* (%)	Outcome Yes *n* (%)	Outcome No *n* (%)	Crude OR (95% CI)	Adjusted OR† (95% CI)	*P*-value
*H. pylori* exposure at year 3						
No	334 (59.8)	13 (3.9)	321 (96.1)	1	1	0.88
Yes	225 (40.3)	10 (4.4)	215 (95.6)	1.15 (0.49, 2.67)	1.07 (0.45, 2.57)	
Cross-sectional analysis of rhinitis at age 5						
*H. pylori* exposure at age 5 (*N* = 847)						
No	477 (56.3)	18 (3.8)	459 (96.2)	1	1	0.86
Yes	370 (43.7)	13 (3.5)	357 (96.5)	0.93 (0.45, 1.92)	0.94 (0.45, 1.94)	
*H. pylori* exposure at ages 3 and 5 (*N* = 593) ‡						
Never infected	222 (37.4)	9 (4.1)	213 (96.0)	1	1	0.83
Infected at age 3 but not at age 5	101 (17.0)	3 (3.0)	48 (97.0)	0.72 (0.19, 2.74)	0.73 (0.19, 2.76)	
Infected at age 5 but not at age 3	126 (21.3)	3 (2.4)	123 (97.6)	0.56 (0.15, 2.18)	0.60 (0.16, 2.26)	
Persistent exposure	144 (24.3)	6 (4.2)	138 (95.8)	1.03 (0.36, 2.96)	1.05 (0.36, 3.03)	

* Reduced numbers as longitudinal analysis performed only on those reporting never rhinitis at age 3.

† ORs adjusted for child's gender, area of residence, and maternal education.

‡ Reduced numbers as *H. pylori* measured on subsample only at age 3.

**Table 6:** Longitudinal and cross-sectional analyses of sensitization in relation to exposure to *Helicobacter pylori* infection

Longitudinal analysis of incident sensitization between ages 3 and 5 (*N* = 552)*	Overall *N* (%)	Outcome Yes *n* (%)	Outcome No *n* (%)	Crude OR (95% CI)	Adjusted OR† (95% CI)	*P*-value
*H. pylori* exposure at year 3						
No	318 (57.6)	7 (2.2)	311 (97.8)	1	1	0.72
Yes	234 (42.4)	6 (2.6)	228 (97.4)	1.17 (0.39, 3.53)	1.23 (0.40, 3.37)	
Cross-sectional analysis of sensitization at age 5						
*H. pylori* exposure at age 5 (*N* = 854)						
No	479 (56.1)	14 (2.9)	465 (97.1)	1	1	
Yes	375 (43.9)	3 (0.8)	372 (99.2)	0.27 (0.08, 0.94)	0.26 (0.07, 0.92)	0.02
*H. pylori* exposure at ages 3 and 5 (*N* = 597)‡						
Never infected	224 (37.5)	7 (3.1)	217 (96.9)	1	1	0.36
Infected at any age up to year 5	373 (62.5)	7 (1.9)	366 (98.1)	0.59 (0.21, 1.71)	0.61 (0.21, 1.76)	

* Reduced numbers as longitudinal analysis performed only on those reporting never sensitization at age 3.

† ORs adjusted for child's gender, area of residence, and maternal education.

‡ Reduced numbers as *H. pylori* measured on subsample only at age 3.

### Effects of *H. pylori* infection on prevalence of allergic outcomes at age 5

The analysis of the four-level *H. pylori* infection variable representing timing of infection revealed the lowest prevalence of the outcomes amongst children infected at age 3 but not at 5, and consistently reduced ORs in infected children for eczema and sensitization, but overall associations did not reach statistical significance (Tables 6). The exposure variable representing current *H. pylori* infection (at age 5) was inversely associated with all outcomes at age 5 except wheeze and reached statistical significance for sensitization (adjusted OR, 95% CI, 0.26; 0.07, 0.92, *P* = 0.02; Table 6). Further adjustment for potential confounders, including childhood and household characteristics, did not materially alter these cross-sectional associations.

## Discussion

This study provides important insights into the role of *H. pylori* in relation to allergic disease and sensitization. As with our study at year 3 follow-up [14], this study demonstrates evidence of a protective effect of *H. pylori* on eczema and sensitization in young children in a low-income birth cohort in which confounding by social advantage or antibiotic therapy is unlikely to play a role. In the longitudinal analysis, infection with *H. pylori* was associated with a 69% decreased risk of incident eczema, and in the cross-sectional analysis, current exposure to *H. pylori* infection at year 5 was associated with a 74% decreased risk of sensitization. However, no significant associations, cross-sectionally or longitudinally, were seen for wheeze and rhinitis, and the directions of the ORs were inconsistent.

The use of a low-income setting prospective birth cohort in assessing the hypothesis that *H. pylori* infection protects against wheeze and allergic diseases has several strengths. The prospective design provides information on temporal relations between the exposure and outcome, hence reverse causation is unlikely to have played a role in this study. Furthermore, the birth cohort design meant it was possible to explore the timing of *H. pylori* exposure, and association with allergic diseases, which has barely been explored in most previous studies. The study also demonstrated very good retention of the surviving mother–child dyads up to the age of 5 years, with less than 6% lost to follow-up between birth and five. *H. pylori* was measured objectively using stool antigen testing, a reliable epidemiological method to determine current infection status in young children with sensitivity of 90% and specificity of 93% [27], although we were unable to specifically measure the strains of *H. pylori*. Other studies have shown protective associations to be particularly strong for the more virulent CagA+ *H. pylori* strains [28]. The prevalence of *H. pylori* infection was higher than that observed in more economically developed countries [29], with a small increase in incident *H. pylori* infections with ageing as expected.

Our findings, however, must be interpreted in the light of several limitations. Whilst the sensitization outcome was measured objectively, measures of wheeze, eczema and rhinitis were based on maternal questionnaire reporting, and hence are susceptible to reporting or recall bias. This is, however, based on the widely validated ISAAC symptoms questionnaire [30], which has been successfully used by our group in under five children [31,32], and in older age groups [2,26]. Moreover, incidence and prevalence of the study outcomes appeared to be positively related to sensitization; albeit weakly with wheeze, and with maternal and paternal allergy, suggesting that some of these are markers of allergy. Even though validated symptom based-questionnaires were used, as these are most practical for a population-based epidemiological study [33], the lack of objective measures of asthma is a limitation. The implications of poor recall on the findings are that some incident cases may have been missed if symptoms only occurred early in the 1- to 3-year follow-up period and did not persist up to the age of 5, although such non-persistent cases are likely to be those with mild disease. Multiple testing might be an issue; however, the consistency of both cross-sectional and longitudinal analyses at a range of time-points makes the possibility that these are chance findings unlikely. A further limitation of the study is that some analyses, particularly longitudinal analyses, suffered from low numbers in some groups which resulted in some wide confidence intervals and hence imprecision of effect estimates. We have only tested a subsample of children at age 3 (*n* = 616 children of 878 available children at age 3) and a complete sample of children at age 5. The implication is that this might have introduced a degree of bias in relation to the outcomes, although this bias is likely to be non-differential. Moreover, we have no data on *H. pylori* status of these children younger than 3 years and hence were unable to assess the prevalence of infection in the first year of life as well as associations with our outcomes. Finally, the presence of *H. pylori* infection may simply be a marker for other microbes or poor hygiene practices that protect against atopic disease [34,35] and hence the associations that we report are not a direct biological effect of the present of the *H. pylori* bacterium itself.

Most studies investigating the link between *H. pylori* infection and asthma and allergic disease to date have been cross-sectional or case–control in design [9]. Studies in young children, particularly those from low-income countries, are remarkably scarce, with only few studies reporting links in children [5,6]. In this study, we have found a significant inverse association between early-life exposure to *H. pylori* infection and the risk of incident eczema between ages 3 and 5 (adjusted OR = 0.31). This finding is in agreement with our previous study in the same cohort [14] and other available cross-sectional studies in children [5,6]. Amongst the US-based studies in children (age 3–19 years), the National Health and Nutrition Examination Survey (NHANES IV) reported significant inverse association between *H. pylori* seropositivity and eczema (adjusted OR = 0.73) [5]. Our findings are also consistent with data reported by Herbarth et al. [6] from Germany who found a 63% reduction in doctor-diagnosed eczema in children (mean age 6.3 years) infected to *H. pylori*. The difference in size of the ORs may be due to variation in age, outcome ascertainment, level of infection (e.g. the prevalence of *H. pylori* in these studies was < 10% [5,6] compared with > 40% in ours), and differences in measurement of infection status (serology vs. rapid stool antigen test used in the current study).

This study also provides some evidence, although cross-sectional, for an inverse association of *H. pylori* with sensitization at age 5 (adjusted OR = 0.26). Most of the available observational studies in children did not specifically explore the effects of *H. pylori* on sensitization [5,6]. In a study comparing Finnish and Russian children, the prevalence of atopic sensitization was higher in Finland than Russia, and it appeared that this was due to an inverse association with *H. pylori* infection [12]. Even though it is difficult to draw direct inference from studies in adults, our findings fit with cross-sectional studies in these age groups [7,10,13]. The NHANES III survey in the United States demonstrated an inverse association with sensitization to pollen and moulds, with a larger association seen in younger subjects (median age < 43 years) and those infected with CagA^+^ strains (OR = 0.69) [7]. However, the same group of investigators in another study reported no association between *H. pylori* infection and serum IgE [13].

One explanation for these findings might be reverse causation, through use of antibiotics, in that use for asthma or allergy may in turn affect *H. pylori* infection. Even though this remains a possibility, in the current study, in children from a low-income cohort with limited access to standard antibiotics and no *H. pylori* eradication programme, this was less likely to be a source of bias. Although reverse causation may be an issue in the cross-sectional analyses, replication of findings in the longitudinal analyses excludes this possibility. *H. pylori* status might also be a proxy indicator of other infections or socio-economic conditions [6,36]. To explore such a possibility, the findings were controlled for markers of socio-economic status, which may have confounded the previous study [8], and adjusted for other infections including geohelminth infections, and commensal bacteria, but no evidence was found to support this argument.

The study did not detect an association of *H. pylori* with wheeze or rhinitis in this cohort of young children on either cross-sectional or longitudinal analyses. One previous study in children, the NHANES IV study, showed reduction in ever having had asthma (OR = 0.69) or allergic rhinitis (OR = 0.60) amongst *H. pylori* infected subjects [5]. Another US-based study in adults also reported inverse associations, and it appeared that the associations were stronger in younger adults (median age < 43 years), for asthma and rhinitis cases with onset during childhood (≤ 15 years), and in those infected by CagA^+^ strains [7]. The lack of associations with reported wheeze and rhinitis in this study may be due to mechanisms other than those related to wheeze or asthma physiopathology, or to outcomes partly unrelated to allergic phenotype [37,38]. Another explanation may be that these observations relate mainly to *H. pylori* CagA^+^ strains, as these have been reported to have strong effects on asthma [7,9]. We have no data on CagA serology; however, a previous study in dyspeptic Ethiopian patients detected CagA genes in 79% of the study subjects [39], suggesting this may be the dominant strain in the population, and perhaps also explaining the strong protective associations seen in the study.

Plausible mechanisms in support of these observations also exist. Studies have shown that the *H. pylori*-induced inflammatory response is associated with Th1-mediated cellular responses [28,40], with higher expression of interferon-γ (IFN-γ) [40], IL-10, and IL-12 [41]. Particularly strong effects were seen in those possessing the pathogenic CagA^+^ strains [28,41]. The protective effects of *H. pylori* against allergy are also mediated by secretion of regulatory T cells (Tregs) [42] that suppress immunity and inflammation via bystander effects of IL-10 [42].

In conclusion, our data are consistent with the hypothesis that *H. pylori* has a protective role against allergy, particularly eczema and sensitization, although further study is needed to establish causality. Investigations of the immunological mechanisms involved may enhance our understanding of the interactions between the gut microbiome and allergic disease, possibly allowing therapeutic manipulation of these pathways in the future in the primary prevention or treatment of these conditions.

## Supporting Information

**Table S1.** Potential and *a priori* confounders measured at an early age and at the age of 5 in relation with symptom outcomes at the age of 5 years. **Table S2.** Potential and *a priori* confounders measured at an early age and at the age of 5 in relation with sensitization at the age of 5 years. **Table S3.** Potential and *a priori* confounders measured at an early age and at the age of 5 in relation with *H. pylori* status at the age of 5 years.
