# Supplementary material for: Exposure to Helicobacter pylori infection in early childhood and the risk of allergic disease and atopic sensitization: a longitudinal birth cohort study
Source: Clin Exp Allergy. 2014 Mar 26;44(4):563–71. doi: 10.1111/cea.12289 (PMC4164268; doi:10.1111/cea.12289)
Supplement: Supplementary file 1 [file cea0044-0563-SD1.doc]

**Online supplement**

**Exposure to *Helicobacter pylori* infection in early childhood and the risk of allergic disease and atopic sensitization: a longitudinal birth cohort study**

Alemayehu Amberbir^1, 2*^, Girmay Medhin^3^, Woldaregay E Abegaz^3^, Charlotte Hanlon^4^, Karen Robinson^5^, Andrew Fogarty^2^, John Britton^2^, Andrea Venn^2^, Gail Davey^6^

**Running title:** *Helicobacter pylori* infection and risk of allergic disease

1. Department of Infectious Disease Epidemiology, London School of Hygiene and Tropical Medicine, UK.
2. Division of Epidemiology and Public Health, University of Nottingham, UK.
3. Aklilu Lemma Institute of Pathobiology, Addis Ababa University, Addis Ababa, Ethiopia.
4. Department of Psychiatry, Addis Ababa University, Addis Ababa, Ethiopia.
5. Centre for Biomolecular Sciences, University of Nottingham, UK.
6. Brighton & Sussex Medical School, University of Brighton, UK.

*Corresponding author, Email: [alemayehu.amberbir@lshtm.ac.uk](mailto:alemayehu.amberbir@lshtm.ac.uk)

London School of Hygiene and Tropical Medicine, Keppel Street, WC1E 7HT, London, UK

Table E1 potential and *a priori* confounders measured at an early age and at the age of 5 in relation with symptom outcomes at the age of 5 years

| Variables | Overall  N (%) | Wheeze  Yes n (%) | Wheeze | Eczema  Yes n (%) | Eczema | Hay fever  Yes n (%) | Hay fever |
| --- | --- | --- | --- | --- | --- | --- | --- |
|  |  |  | Crude OR  (95% CI) |  | Crude OR  (95% CI) |  | Crude OR  (95% CI) |
| Urban area of residence | 105 (12.3) | 5 (4.8) | 1.12 (0.43,2.94) | 4 (3.8) | 0.88 (0.31,2.56) | 3 (2.9) | 0.76 (0.23,2.53) |
| Male gender | 434 (50.9) | 23 (5.3) | 1.61(0.82,3.19) | 15 (3.5) | 0.68 (0.34,1.33) | 18 (4.2) | 1.35 (0.65,2.79) |
| Maternal education (formal vs. non formal) | 160 (18.8) | 7 (4.4) | 1.01 (0.45,2.34) | 5 (3.1) | 0.69 (0.26,1.80) | 6 (3.8) | 1.04 (0.42,2.58) |
| Maternal history of allergy | 55 (6.5) | 9 (16.4) | 5.37 (2.37,12.18) | 9 (16.4) | 5.58 (2.45,12.69) | 9 (16.4) | 6.89 (2.96,16.04) |
| Paternal history of allergy | 39 (4.6) | 3 (7.7) | 1.90 (0.56,6.52) | 7 (18.0) | 5.91 (2.38,14.67) | 5 (12.8) | 4.45 (1.60,12.39) |
| Breast feeding at yr 3 | 91 (10.7) | 5 (5.5) | 1.32 (0.50,3.47) | 4 (4.4) | 1.04 (0.36,3.02) | 2 (2.2) | 0.56 (0.13,2.41) |
| Household size |  |  |  |  |  |  |  |
| 1-6 | 484 (56.8) | 22 (4.6) | 1 | 18 (3.7) | 1 | 13 (2.7) | 1 |
| 7^+^ | 368 (43.2) | 15 (4.1) | 0.89 (0.46,1.75) | 18 (4.9) | 1.33 (0.68,2.60) | 18 (4.9) | 1.86 (0.90,3.86) |
| No of older siblings |  |  |  |  |  |  |  |
| 0 | 122 (14.3) | 6 (4.9) | 1 | 2 (1.6) | 1 | 4 (3.3) | 1 |
| 1-3 | 463 (54.3) | 20 (4.3) | 0.87 (0.34,2.23) | 22 (4.8) | 2.99 (0.69,12.96) | 13 (2.8) | 0.85 (0.27,2.66) |
| 4-10 | 267 (31.3) | 11 (4.1) | 0.83 (0.30,2.30) | 12 (4.5) | 2.82 (0.62,12.89) | 14 (5.2) | 1.63 (0.52,5.08) |
| Child’s sleeping place |  |  |  |  |  |  |  |
| Bed | 44 (5.2) | 1 (2.3) | 1 | 1 (2.3) | 1 | 1 (2.3) | 1 |
| Mattress | 198 (23.2) | 13 (6.6) | 3.02 (0.38,23.95) | 11 (5.6) | 2.53 (0.32,20.28) | 8 (4.0) | 1.81 (0.23,14.95) |
| Grass matting | 610 (71.6) | 23 (3.8) | 1.68 (0.22,12.80) | 24 (3.9) | 1.76 (0.23,13.36) | 22 (3.6) | 1.61 (0.21,12.25) |
| Insecticide use in the home | 570 (66.9) | 24 (4.2) | 0.91 (0.46,1.82) | 25 (4.4) | 1.13 (0.55,2.33) | 21 (3.7) | 1.04 (0.48,2.24) |
| Indoor cooking | 630 (73.9) | 30 (4.8) | 1.54 (0.66,3.55) | 27 (4.3) | 1.06 (0.49,2.29) | 21 (3.3) | 0.73 (0.34,1.58) |
| Indoor charcoal use | 160 (18.8) | 7 (4.4) | 1.01 (0.44,2.34) | 4 (2.5) | 0.53 (0.18,1.52) | 8 (5.0) | 1.53 (0.67,3.49) |
| Smoking in the house | 103 (12.1) | 4 (3.9) | 0.88 (0.30,2.53) | 2 (1.9) | 0.42 (0.10,1.76) | 3 (2.9) | 0.77 (0.23,2.59) |
| Antibiotic use | 120 (14.1) | 7 (5.8) | 1.45 (0.62,3.38) | 10 (8.3) | 2.47 (1.16,5.28) | 8 (6.7) | 2.20 (0.96,5.06) |
| Thatched roof vs. corrugated iron sheet | 648 (77.8) | 26 (4.0) | 0.66 (0.32,1.37) | 30 (4.6) | 1.45 (0.59,3.54) | 23 (3.6) | 0.94 (0.39,2.22) |

*Area of residence and maternal education were measured during pregnancy, child’s gender at birth, breastfeeding at age 3 and the rest were measured at age 5

Table E2 potential and *a priori* confounders measured at an early age and at the age of 5 in relation with sensitization at the age of 5 years

| Variables | Overall  N (%) | Sensitization  Yes n (%) | Sensitization |
| --- | --- | --- | --- |
|  |  |  | Crude OR  (95% CI) |
| Urban area of residence | 104 (12.2) | 2 (1.9) | 0.96 (0.22,4.27) |
| Male gender | 434 (50.8) | 9 (2.1) | 1.09 (0.42,2.86) |
| Maternal education (formal vs. non formal) | 162 (19.0 ) | 2 (1.2) | 0.57 (0.13,2.50) |
| Maternal history of allergy | 55 (6.5) | 3 (5.6) | 3.26 (0.91,11.7) |
| Paternal history of allergy | 38 (4.5) | 2 (5.3) | 2.93 (0.64,13.4) |
| Breast feeding at yr 3 | 93 (10.6) | 1 (1.1) | 0.52 (0.07,3.98) |
| Household size |  |  |  |
| 1-6 | 479 (56.7) | 10 (2.1) | 1 |
| 7^+^ | 366 (43.3) | 7 (1.9) | 0.91 (0.34,2.43) |
| No of older siblings |  |  |  |
| 0 | 120 (14.2) | 2 (1.7) | 1 |
| 1-3 | 459 (54.3) | 11 (2.4) | 1.45 (0.32,6.64) |
| 4-10 | 266 (31.5) | 4 (1.5) | 0.90 (0.16,5.00) |
| Child’s sleeping place |  |  |  |
| Bed/mattress | 240 (28.4) | 6 (2.5) | 1 |
| Grass matting | 605 (71.6) | 11 (1.8) | 0.72 (0.26,1.98) |
| Insecticide use in the home | 564 (66.8) | 13 (2.3) | 1.63 (0.53, 5.06) |
| Indoor cooking | 624 (73.9) | 12 (1.9) | 0.85 (0.29,2.43) |
| Indoor charcoal use | 159 (18.8) | 3 (1.9) | 0.92 (0.26,3.25) |
| Smoking in the house | 103 (12.1) | 1 (1.0) | 0.44 (0.06,3.40) |
| Antibiotic use | 119 (14.1) | 3 (2.5) | 1.32 (0.37,4.65) |
| Thatched roof vs. corrugated iron sheet | 650 (77.8) | 14 (2.2) | 1.34 (0.38,4.73) |

*Area of residence and maternal education were measured during pregnancy, child’s gender at birth, breastfeeding at age 3 and the rest were measured at age 5

**Table E3 Potential and *a priori* confounders measured at an early age and at the age of 5 in relation with *H. pylori*** status at the age of 5 years

| **Variables** | **Overall**  **N (%)** |  | ***H. pylori* at age 5** | | |
| --- | --- | --- | --- | --- | --- |
|  |  | **Yes**  **(%)** | | **No**  **(%)** | **Crude OR**  **(95% CI)** |
| **Urban area of residence** | **104 (12.1)** | **52 (50.0)** | | **52 (50.0)** | **1.32 (0.87,1.99)** |
| **Male gender** | **434 (50.6)** | **192 (44.2)** | | **242 (55.8)** | **1.02 (0.78,1.34)** |
| **Maternal education (formal vs. non formal)** | **161 (18.8)** | **65 (40.4)** | | **96 (59.6)** | **0.83 (0.59,1.18)** |
| **Maternal history of allergy** | **53 (6.3)** | **24 (45.3)** | | **29 (54.7)** | **1.07 (0.61,1.87)** |
| **Paternal history of allergy** | **38 (4.5)** | **15 (39.5)** | | **23 (60.5)** | **0.83 (0.43,1.62)** |
| **Breast feeding at yr 3** | **90 (10.6)** | **44 (48.9)** | | **46 (51.1)** | **1.25 (0.81,1.94)** |
| **Household size** |  |  | |  |  |
| **1-3**  **4-6** | **24 (2.8)**  **457 (54.0)** | **12 (50.0)**  **187 (40.9)** | | **12 (50.0)**  **270 (59.1)** | **1**  **0.69 (0.30,1.58)** |
| **7^+^** | **366 (43.2)** | **171 (46.7)** | | **195 (53.3)** | **0.88 (0.38,2.01)** |
| **No of older siblings** |  |  | |  |  |
| **0** | **122 (14.4)** | **46 (37.7)** | | **76 (62.3)** | **1** |
| **1-3** | **459 (54.2)** | **200 (43.6)** | | **259 (56.4)** | **1.28 (0.85,1.92)** |
| **4-10** | **266 (31.4)** | **124 (46.6)** | | **142 (53.4)** | **1.44 (0.93,2.24)** |
| **Child’s sleeping place** |  |  | |  |  |
| **Bed** | **43 (5.1)** | **17 (39.5)** | | **26 (60.5)** | **1** |
| **Mattress** | **198 (23.4)** | **95 (48.0)** | | **103 (52.0)** | **1.41 (0.72,2.77)** |
| **Grass matting** | **606 (71.6)** | **258 (42.6)** | | **348 (57.4)** | **1.13 (0.60,2.13)** |
| **Insecticide use in the home** | **566 (66.8)** | **245 (43.3)** | | **321 (56.7)** | **0.95 (0.71,1.27)** |
| **Indoor cooking** | **626 (73.9)** | **264 (42.2)** | | **362 (57.8)** | **0.79 (0.58,1.08)** |
| **Indoor charcoal use** | **159 (18.8)** | **76 (47.8)** | | **83 (52.2)** | **1.23 (0.87,1.74)** |
| **Smoking in the house** | **103 (12.1)** | **49 (47.6)** | | **54 (52.4)** | **1.20 (0.79,1.81)** |
| **Antibiotic use** | **120 (14.1)** | **45 (37.5)** | | **75 (62.5)** | **0.74 (0.50,1.11)** |
| **Thatched roof vs. corrugated iron sheet** | **652 (77.8)** | **276 (42.3)** | | **376 (57.7)** | **0.73 (0.53,1.02)** |

***Area of residence and maternal education were measured during pregnancy, child’s gender at birth, breastfeeding at age 3 and the rest were measured at age 5**
